# Adverse events following HPV vaccine reported to the Vaccine Adverse Event Reporting System

**DOI:** 10.1371/journal.pone.0351652

**Published:** 2026-06-17

**Authors:** Siqi Zhang, Yu Li, Miaomiao Liu, Jiajia Yang, Xiong Wang, Mingshan Wang

**Affiliations:** 1 Department of Urology, Xiang'an Hospital of Xiamen University, School of Medicine, Xiamen University, Xiamen, China; 2 Department of Medical Oncology, Xiang'an Hospital of Xiamen University, School of Medicine, Xiamen University, Xiamen, Fujian, China; 3 Eye Institute & Affiliated Xiamen Eye Center, School of Medicine, Xiamen University, Xiamen, Fujian, China; Federal University Otuoke, NIGERIA

## Abstract

**Background:**

Vaccination against high-risk HPV types is a key preventive measure. However, concerns regarding vaccine safety may hinder vaccination efforts. This study aims to evaluate adverse events (AEs) reported in the Vaccine Adverse Event Reporting System (VAERS) following HPV vaccination from 2006 to 2024, providing insights into its safety profile.

**Methods:**

We analyzed VAERS data, a spontaneous reporting system containing de-identified AE reports. Four disproportionality analyses (ROR, PRR, BCPNN, and MGPS) were applied, and adverse event signals were defined only when the positive criteria were simultaneously met across all four methods. Statistical analyses were performed using R software and Microsoft Excel, with a significance threshold of p < 0.05.

**Results:**

A total of 77,909 HPV vaccine-related AE reports were analyzed, with 68.4% involving females and 48.7% affecting individuals under 18. Serious AEs accounted for 11,659 reports, with headache and fatigue being the most common. Syncope was the most frequent signal, while postural orthostatic tachycardia syndrome (POTS) exhibited the strongest signal strength. Approximately 90% of AEs occurred within 30 days post-vaccination. Among vaccine types, HPV4 had the highest number of reports, and intramuscular injection was the most common administration route.

**Conclusion:**

This study offers an updated pharmacovigilance assessment of adverse events reported following HPV vaccination, highlighting reported patterns and statistical signals that may warrant further investigation.

## Introduction

Human papillomavirus (HPV) is a common sexually transmitted infectious virus and the causative agent of various HPV-related lesions and cancers, such as cervical cancer, penile cancer, vulvar cancer, vaginal cancer and oropharyngeal cancer [1–5]. It is estimated that over 80% of women and over 90% of men will be infected with HPV during their lifetime [6]. Moreover, more than 95% of cervical cancer cases are associated with HPV [7]. Cervical cancer is the fourth most common cancer among women globally, with 661,021 new cases and 348,189 deaths reported in 2022 [8,9]. HPV has multiple types, classified into high-risk and low-risk categories based on their carcinogenic potential [10]. HPV16 and HPV18 are the most common high-risk types, found in approximately 70% of cervical cancer cases [11–14]. Additionally, HPV types 31, 33, 35, 39, 45, 51, 52, 56, 58, and 59 can also cause cervical cancer [15].

In urology, HPV infection is not only associated with common sexually transmitted diseases such as genital warts, but is also closely linked to genitourinary malignancies—most notably penile cancer and its premalignant lesion, penile intraepithelial neoplasia (PeIN). Available evidence suggests that a substantial proportion of penile squamous cell carcinoma and its precursor lesions are associated with persistent infection by high-risk HPV types. Because penile cancer often requires surgery-based multimodal management and can markedly impair urinary function, sexual function, and psychological well-being, vaccination against high-risk HPV types represents an important primary preventive measure.

Identifying HPV types provides opportunities for the prevention and control of HPV-related cancers. Vaccination targeting high-risk HPV types associated with cervical cancer is one of the most effective measures to reduce cancer incidence. Administering the HPV vaccine before exposure can effectively prevent most HPV-related cancers [16,17]. To date, six licensed prophylactic HPV vaccines have been developed: three bivalent vaccines, two quadrivalent vaccines, and one nonavalent vaccine [15]. In 2018, the World Health Organization (WHO) called for action to eliminate cervical cancer as a public health issue, aiming for 90% of girls to be fully vaccinated against HPV by the age of 15 by 2030 and to reduce cervical cancer incidence by 42% by 2045 [18]. Currently, over 60% of countries have included HPV vaccination for girls in their national immunization programs, and more than 20% have extended these programs to include boys [19]. In 2018, the U.S. Food and Drug Administration expanded the approved use of the HPV vaccine to include women and men aged 27–45 [20]. As HPV vaccine coverage increases, pharmacovigilance efforts should be further strengthened.

The Vaccine Adverse Event Reporting System (VAERS) is a critical early warning system for detecting potential safety issues associated with vaccines licensed in the United States [21]. Data from this system play a significant role in shaping public perceptions of vaccine safety [22]. Although several studies have previously evaluated adverse events following HPV vaccination using VAERS data, continuous post-marketing surveillance remains a fundamental component of pharmacovigilance, particularly as vaccination strategies and vaccine formulations evolve over time. The present study extends existing evidence by analyzing VAERS reports over an expanded period from 2006 to 2024, encompassing the widespread real-world use of the 9-valent HPV vaccine. In addition, we applied four complementary disproportionality algorithms and adopted a stringent consensus-based signal definition to enhance robustness and reduce false-positive findings.

## Materials and methods

Given that the VAERS database maintains anonymous and de-identified patient records while being publicly accessible, it typically does not require informed consent or ethical approval.

### Study design and data source

The data for this study originated from reports within the VAERS database spanning from 2006 to 2024. VAERS operates as a spontaneous reporting system, gathering adverse event reports from across the globe [23]. The reports primarily include demographic data of the vaccine recipients, information about the administered vaccine, and the AEs that occurred after vaccination. This study employs the preferred term (PT) from the MedDRA drug adverse reaction terminology set. The international standardization of AE descriptions involves using MedDRA [23,24]. Its hierarchical structure allows PTs to be classified into relevant system organ class, which represents the highest level in MedDRA’s hierarchy. According to the definition in the Federal Regulations, the reports are categorized as serious and non-serious reports. Events such as death, life-threatening diseases, hospitalization or prolonged existing hospitalization, and permanent disability are classified as serious events [25]. Reports submitted by vaccine manufacturers require a separate follow-up procedure, while cases reported by others are asked to provide follow-up medical records for further review [23]. Additionally, we assessed the occurrence timing of specific adverse events induced by HPV vaccine, excluding cases with missing or incorrect dates.

### Statistical analysis

**Fig 1:**
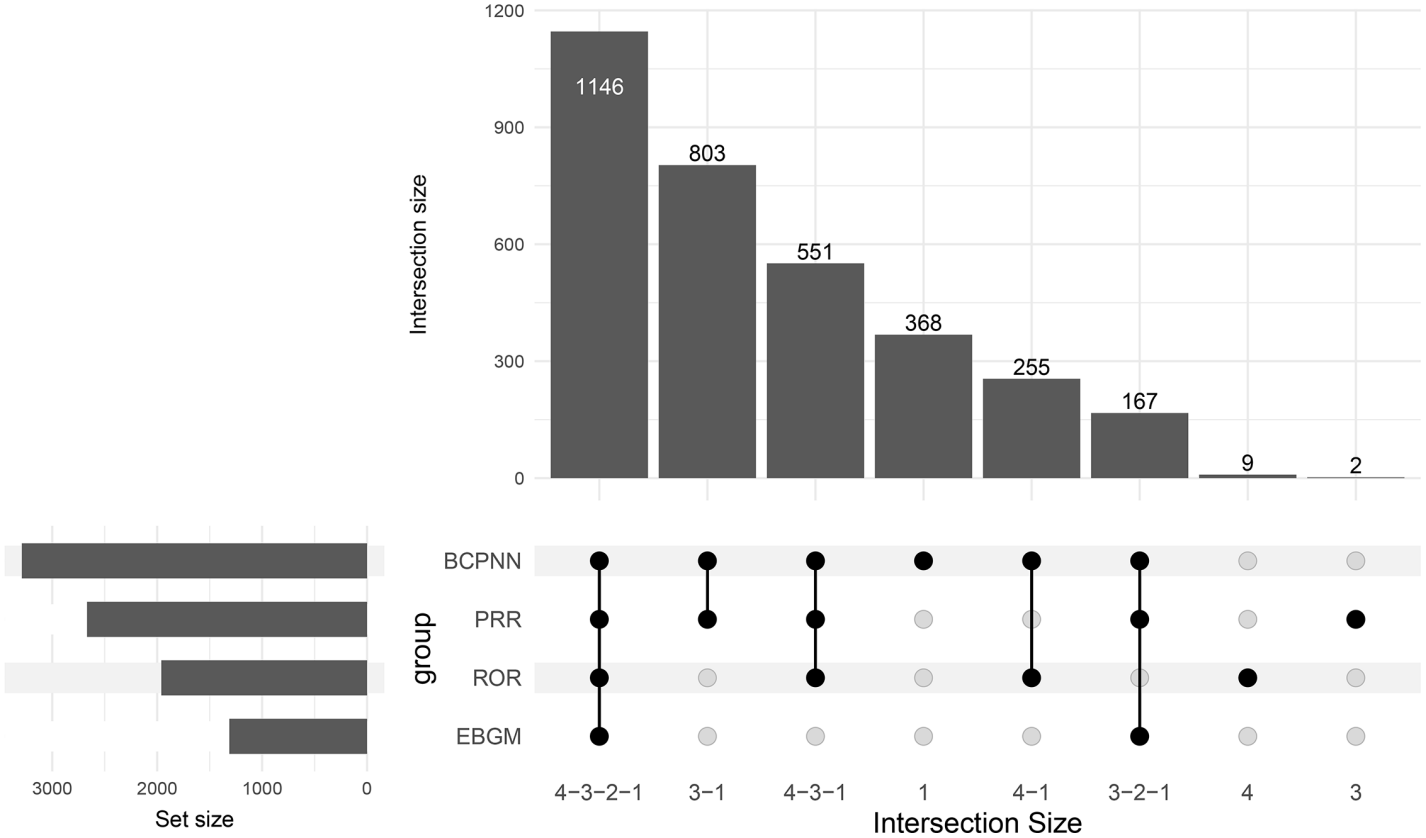
Signal detection results using ROR, PRR, BCPNN, and MGPS.

For disproportionality analyses, the database consisted of all adverse event reports associated with vaccines other than HPV vaccines recorded in the VAERS database during the same study period (2006–2024). Specifically, for each preferred term (PT), a 2 × 2 contingency table was constructed as follows: (a) reports of the specific PT following HPV vaccination; (b) reports of all other PTs following HPV vaccination; (c) reports of the same PT following non-HPV vaccinations; and (d) reports of all other PTs following non-HPV vaccinations. All reports included in the database were extracted from the same VAERS time window to ensure temporal consistency and comparability between HPV and non-HPV vaccine reports. The disproportionality analysis is frequently employed to investigate potential correlations between drugs and adverse events within post-market surveillance databases [26]. In our study, disproportionality analyses using Reporting Odds Ratio (ROR) [27], Proportional Reporting Ratio (PRR) [28], Bayesian Confidence Propagation Neural Network (BCPNN) [29] and Multi-Item Gamma Poisson Shrinker (MGPS) [30] were employed to identify potential AE signals related to HPV vaccine. This was done to confirm our findings and reduce false positive safety signals. The detailed formulas and criteria for these four algorithms are provided in (Tables 1 and 2). In our study, to avoid false-positive safety signals, an adverse event must meet the positive criteria of all four algorithms simultaneously (Fig 1). The larger the ROR value, the stronger the signal, indicating a more pronounced correlation between the HPV vaccine and AE. Descriptions of count data were presented using case numbers and proportions. Data processing and statistical analysis were conducted using R software (4.3.3) and Microsoft Excel 2021. P value less than 0.05 was considered significant.

**Table 1:** Four-grid table of signal detection.

project	Target Adverse Events	Other Adverse Events	Total
target drug	a	b	a + b
Other drugs	c	d	c + d
Total	a + c	b + d	N = a + b + c + d

A contingency table for the calculation formula of the disproportionality analysis.

**Table 2:** Four major algorithms used to assess potential associations between HPV vaccine and AEs.

Algorithms	Equation	Criteria
ROR	ROR=ad/b/c 95%CI=eln(ROR)±1.96(1/a+1/b+1/c+1/d)^0.5	lower limit of 95% CI > 1, N ≥ 3
PRR	PRR=a(c+d)/c/(a+b) χ2=[(ad−bc)^2](a+b+c+d)/[(a+b)(c+d)(a+c)(b+d)]	PRR ≥ 2, χ2 ≥ 4, N ≥ 3
BCPNN	$IC={\text{log}}_{\text{2}}a(a+b+c+d)/((a+c)(a+b))$	IC025 > 0
MGPS	EBGM=a(a+b+c+d)/(a+c)/(a+b) 95%CI=eln(EBGM)±1.96(1/a+1/b+1/c+1/d)^0.5	EBGM05 > 2

Abbreviation: ROR, Reporting Odds Ratio; PRR, Proportional Reporting Ratio; BCPNN, Bayesian Confidence Propagation Neural Network; MGPS, Multi-item Gamma Poisson Shrinker; EBGM, Empirical Bayesian Geometric Mean; CI, Confidence Interval; χ2, Chi-square; IC, Information Component; IC025, the lower limit of the 95% one-sided confidence interval for IC; EBGM05, the lower limit of the 95% CI for EBGM.

## Result

### Descriptive analysis

Our study included a total of 77,909 AE reports related to the HPV vaccine (Table 3), with California being the region with the highest number of reports, totaling 5,030 HPV vaccine-related AEs. Individuals under 18 years of age were the primary group affected by HPV vaccine-related AEs, with 37,917 cases. Additionally, females accounted for 68.4% of the total HPV vaccine-related AEs. Among these reports, there were 11,659 serious HPV vaccine-related AEs, and 60,664 cases involved the use of the HPV vaccine alone. Most patients who experienced AEs after receiving the HPV vaccine recovered; however, it is noteworthy that hospitalization was the most common outcome among serious AEs. (Table 4) shows the 20 most common PTs in serious reports, with Headache and Fatigue being the most frequently reported, with 2,772 (2.7%) and 2,065 (2.0%) occurrences, respectively.

**Table 3:** Baseline data for patients using HPV vaccine reported in the VAERS database.

Characteristic	N	Percentage
**Total**	77909	
**State**		
Foreign state	19005	24.40%
California	5030	6.50%
Texas	3138	4.00%
Pennsylvania	2975	3.80%
New York	2737	3.50%
**Age**		
<18	37917	48.70%
18-64	16193	20.80%
65-84	74	0.10%
≥85	4	0.00%
Unknown	23721	30.40%
**Sex**		
Female	53301	68.40%
Male	10951	14.10%
Unknown	13657	17.50%
**Serious symptom**		
No	66250	85.00%
Yes	11659	15.00%
**Alone medication**		
No	17245	22.10%
Yes	60664	77.90%
**Outcome**		
Recovered	26287	33.70%
Hospitalization	7891	10.10%
Disable	3808	4.90%
Life threat	1183	1.50%
Died	641	0.80%
Prolonged hospitalization	312	0.40%

**Table 4:** Most frequent MedDRA preferred terms for serious following HPV vaccine, 2006–2024.

PT	N	Percentage
Headache	2772	2.7%
Fatigue	2065	2.0%
Dizziness	1925	1.8%
Nausea	1494	1.4%
Pyrexia	1240	1.2%
Syncope	1229	1.2%
Pain	1207	1.2%
Arthralgia	1167	1.1%
Asthenia	1060	1.0%
Malaise	1015	1.0%
Pain in extremity	954	0.9%
Muscular weakness	924	0.9%
Hypoaesthesia	889	0.9%
Vomiting	877	0.8%
Abdominal pain	870	0.8%
Dyspnoea	860	0.8%
Paraesthesia	776	0.7%
Myalgia	750	0.7%
Loss of consciousness	622	0.6%
Gait disturbance	609	0.6%

Additionally, we compiled the vaccine information from these reports (Table 5). Among the three vaccine types, the HPV4 vaccine had the highest number of AE reports, with 47,451 cases. The vast majority of vaccines were produced by Merck & Co. Inc., accounting for approximately 90.20% of all vaccine manufacturers. Regarding the number of doses, approximately 33.30% of the reports involved a single dose, with the majority of reports involving up to three doses. Nearly half of the vaccine administration routes were intramuscular injections (44.80%), and the most common injection sites were the left arm and right arm.

**Table 5:** Vaccine information on reports of HPV vaccine-related adverse events in the VAERS database, 2006–2024.

Variable	N	Percentage
**Type of vaccine**		
HPV2	4903	6.30%
HPV4	47451	60.50%
HPV9	23247	29.70%
Unknown	2766	3.50%
**Vaccine manufacturers**		
Glaxosmithkline biologicals	4903	6.30%
Merck & Co. Inc.	70698	90.20%
Unknown manufacturer	2766	3.50%
**Dose of vaccine**		
1	26071	33.30%
2	13871	17.70%
3	9213	11.80%
4	558	0.70%
5	30	0.00%
6	18	0.00%
7+	18	0.00%
Unknown	28588	36.50%
**Route of vaccination**		
Intramuscular injection	35133	44.80%
Syringe	2428	3.10%
Other ways	3158	4.00%
Subcutaneous injection	357	0.50%
Injection, site not specified	326	0.40%
**Vaccination site**		
Left arm	15770	20.10%
Right arm	10730	13.70%
Left leg	304	0.40%
Right leg	247	0.30%
Gluteus medius	168	0.20%
**Vaccine name**		
HPV (Cervarix)	4903	6.30%
HPV (Gardasil 9)	23247	29.70%
HPV (Gardasil)	47451	60.50%
HPV (No brand name)	2766	3.50%

### Signal of preferred terms

**Fig 2:**
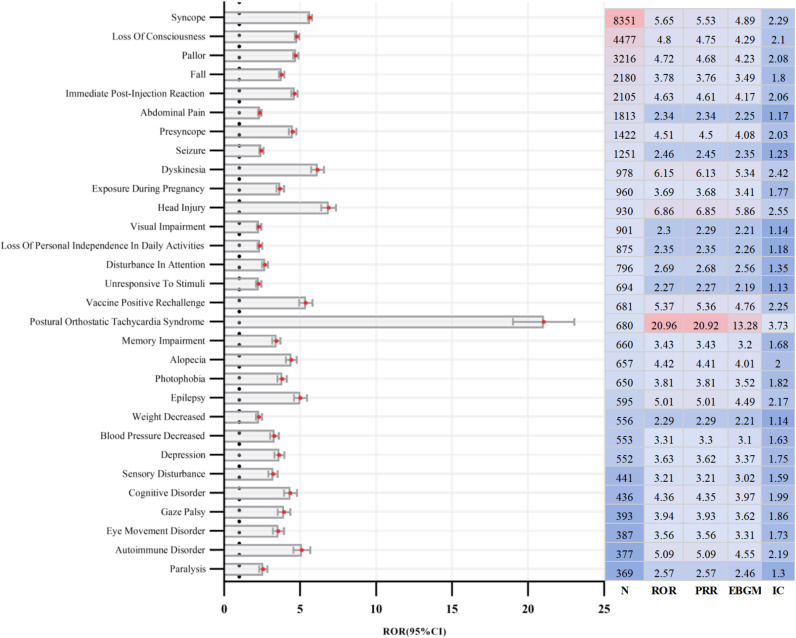
The top 30 preferred terms by frequency.

Through signal screening, a total of 1,146 positive signals were identified (S1 Table), with the most frequent PT being syncope (n = 8,351), followed by loss of consciousness, pallor, fall, immediate post-injection reaction, abdominal pain, presyncope, seizure, dyskinesia, and exposure during pregnancy (Fig 2). Additionally, among the top 30 most frequent occurrences, although postural orthostatic tachycardia syndrome (POTS) exhibited the highest signal strength based on disproportionality metrics, its absolute reporting frequency (n = 680) was lower than that of more common events.

### Time-to-onset analysis

**Fig 3:**
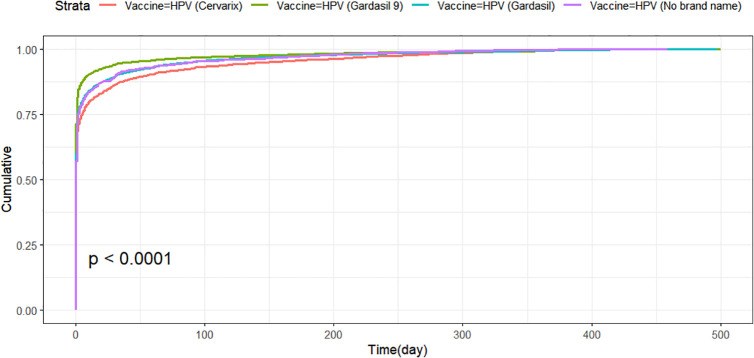
Time-to-onset distribution of adverse events.

After excluding reports with missing AE onset time, we analyzed the onset time of HPV vaccine-related AEs and compared the onset times of AEs for four different HPV vaccine brands (Fig 3). The results showed that approximately 90% of AEs occurred within 0–30 days after vaccination. Among the four vaccines, the shortest AE onset time was observed for HPV (Gardasil 9), while the longest AE onset time was seen for HPV (Cervarix).

## Discussion

This study conducted a comprehensive analysis of adverse event reports following HPV vaccination using the VAERS database from 2006 to 2024. The findings provide important insights into the safety profile of the HPV vaccine and highlight areas that require further investigation.

First, most AE reports came from female recipients under 18, which aligns with the target population of national HPV vaccination programs [18]. Second, although the majority of reported adverse events were non-serious and self-resolving, the significant proportion of severe adverse events (such as hospitalization) emphasizes the importance of ongoing monitoring and follow-up. Notably, headache and fatigue were the most common severe adverse events, consistent with previous vaccine safety studies [31]. Third, signal detection analysis revealed that syncope, loss of consciousness, and pallor were the most common PTs, while postural orthostatic tachycardia syndrome (POTS) exhibited the strongest signal strength. The identification of a disproportional reporting signal for POTS does not imply a causal relationship but indicates a reporting pattern that has been previously noted in passive surveillance systems and warrants cautious interpretation [32]. In spontaneous reporting systems, a high disproportionality measure for a rare event does not necessarily translate into a large clinical or public health impact, particularly when the absolute number of reports is low.

Importantly, findings from spontaneous reporting systems such as VAERS should be interpreted within the broader hierarchy of evidence. While randomized controlled trials and large cohort studies provide more robust estimates of incidence and risk, passive surveillance systems play a complementary role by enabling the detection of rare, unexpected, or population-specific reporting patterns that may not be captured in pre-licensure trials. In this context, the signals identified in the present study largely reflect adverse events that are already well characterized in higher-quality studies, supporting the overall consistency of the safety profile observed across different study designs [33]. However, analyzing VAERS data only assesses the risk of experiencing specific adverse events in individuals who have had an adverse reaction, and does not imply a causal relationship between HPV vaccination and adverse events in the general population. This study further supports the need for large-scale epidemiological studies to clarify causal relationships. Additionally, the analysis of onset time shows that most adverse events occurred within 30 days of vaccination, which is consistent with patterns observed in other vaccine safety studies [33].

Our findings are broadly consistent with previous VAERS-based analyses of HPV vaccine safety, which have reported syncope, headache, and fatigue as commonly reported adverse events following vaccination. The prominence of syncope-related signals is biologically plausible and aligns with well-recognized vasovagal responses among adolescents, particularly in the context of injection-related anxiety. While no fundamentally new safety concerns were identified, the persistence of certain signals over time underscores the value of continuous surveillance and updated analyses as vaccination programs evolve.Syncope was the most frequently reported adverse event signal in this study. This finding is consistent with evidence from large observational studies and post-licensure surveillance indicating that syncope following vaccination is a well-recognized, transient physiological response, particularly among adolescents and young adults. Previous studies have demonstrated that post-vaccination syncope is often related to anxiety, pain, or vasovagal reactions rather than vaccine-specific toxicity. As a result, current immunization guidelines recommend post-vaccination observation periods for adolescents to reduce the risk of injury associated with fainting. The high reporting frequency of syncope observed in the VAERS database therefore aligns with established clinical knowledge and does not indicate a novel safety concern. Headache and fatigue were among the most commonly reported adverse events in serious reports. These symptoms have been consistently documented in randomized controlled trials and meta-analyses of HPV vaccines, where they are typically classified as mild to moderate, self-limiting systemic reactions. Large pre-licensure clinical trials and post-marketing cohort studies have shown that the incidence of headache and fatigue following HPV vaccination is comparable to that observed with other adolescent vaccines and reflects expected immune-mediated responses rather than clinically significant adverse outcomes.

The findings of this study highlight the critical role of pharmacovigilance systems, such as VAERS, in maintaining public trust in vaccines. Research has shown that between 2015 and 2018, concerns about HPV vaccine safety among parents who were hesitant about the vaccine increased by approximately 80% [22]. Additionally, due to increased vaccine hesitancy in the past, many countries, such as Denmark and Italy, faced resistance to HPV vaccination [34–37]. From the perspective of public trust, serious AEs significantly affect people’s perception of the safety of the HPV vaccine. Therefore, we have provided the frequency of serious AEs following HPV vaccination [38]. Encouragingly, serious AEs account for only a small proportion of the reported HPV vaccine-related AEs. While the overall safety of the HPV vaccine is favorable, identifying and addressing potential risks is crucial to maintaining high vaccination coverage. Ensuring the safety and efficacy of the HPV vaccine is especially important in global efforts to eliminate cervical cancer. Healthcare providers should pay particular attention to the potential for serious adverse events in high-risk populations.

This study has some inherent limitations due to the use of a spontaneous reporting system. VAERS data may be subject to underreporting, reporting bias, and an inability to establish causality [23,39]. Additionally, the lack of detailed clinical information limits the depth of the analysis. A stratified analysis by vaccine formulation was not performed, which should be considered when interpreting the findings. However, this study includes large-scale data spanning nearly two decades, providing a solid foundation for signal detection and trend analysis. The use of multiple disproportionality analysis methods further strengthens the reliability of the results. Fourth, we did not perform disproportionality analyses stratified by individual HPV vaccine formulation (e.g., HPV2, HPV4, HPV9 separately). While such stratification could provide formulation-specific safety insights, the reduced report counts for several adverse events after stratification would likely produce unstable or spurious signals. Readers should therefore interpret the combined signals reported here as representing the aggregate HPV vaccine class, recognizing that individual formulations may have different reporting patterns. Future studies using larger active surveillance systems or pooled data sources should address formulation-specific safety profiles. Readers should therefore interpret the combined signals reported here as representing the aggregate HPV vaccine class, recognizing that individual formulations may have different reporting patterns.

From a public health perspective, it is essential to emphasize that the findings of this study should be interpreted within the broader context of HPV vaccination safety and effectiveness. Although multiple statistical signals were identified through disproportionality analyses, the vast majority of VAERS reports following HPV vaccination were classified as non-serious. Moreover, the identification of a signal in a passive surveillance system does not imply a causal relationship but rather indicates an area for further evaluation using more rigorous study designs. Importantly, extensive evidence from randomized controlled trials, large-scale cohort studies, and long-term population-based surveillance has consistently demonstrated that HPV vaccination is highly effective in preventing HPV infection, cervical intraepithelial neoplasia, and HPV-related cancers, with a favorable safety profile. The substantial and well-established benefits of HPV vaccination in reducing cancer incidence and mortality far outweigh the potential, unconfirmed risks suggested by spontaneous reporting systems. In this context, the present analysis should be viewed as a component of ongoing pharmacovigilance efforts aimed at maintaining public trust through transparency and continuous safety monitoring, rather than as evidence challenging the overall safety or public health value of HPV vaccination.

## Conclusion

In conclusion, this study presents a large-scale analysis of adverse event reports following HPV vaccination in the VAERS database and identifies disproportionality signals that contribute to ongoing pharmacovigilance efforts. Ongoing monitoring and transparent communication of vaccine safety information are crucial for maintaining public trust and achieving the global goal of cervical cancer elimination. Overall, these findings support the role of post-marketing surveillance in complementing evidence from clinical trials and epidemiological studies to ensure the safe and effective use of HPV vaccines in public health programs.

## Supporting information

S1 Table The list of the first 100 positive signals. (XLSX)
